# Supplementary material for: Metasurface absorber enhanced thermoelectric conversion
Source: Nanophotonics. 2024 Feb 9;13(8):1361–8. doi: 10.1515/nanoph-2023-0653 (PMC11636509; doi:10.1515/nanoph-2023-0653)
Supplement: Supplementary file 1 — Supplementary Material Details [file j_nanoph-2023-0653_suppl_001.pdf]

Supplementary information

Metamaterial absorber enhanced thermoelectric conversion

Ryosuke Nakayama, Sohei Saito, Takuo Tanaka*, and Wakana Kubo*

Supplementary information

Supplemental text S1, S2
Table S1, S2
Fig. S1-S4

Supplemental text

S1. Calibration of thermistors

A thermistor probe (Micro-BetaCHIP thermistor probe, Measurement Specialties, Inc.) was used to measure environmental temperature. The thermistor is a negative temperature coefficient (NTC) type and has a probe head with a length and diameter of 3.3 and 0.3 mm, respectively. The wires (diameter: 0.15 mm) were connected to the head of the thermistor, which was connected to a temperature sensing circuit that produced an output voltage linear to the environmental temperature.

First, we calibrated thermistors because each thermistor has an intrinsic temperature property. Thermistor 1 and 2 were affixed with Kapton tape to be exposed to the same temperature environment, and we were put in a carbon pod put in an electric furnace set to 100 °C for the measurement. We attach a thermocouple to the thermistors to monitor the environmental temperature as a reference and calibrate thermistors.

The measured temperature can be calculated using

$$\frac{1}{T} = \frac{1}{T_0} + \frac{1}{B} ln \frac{R}{R_0} \qquad \text{Eq. S(1)}$$

where B, R_0, and R represent a beta parameter, resistance of the circuit at the standard temperature (T_0, 298.15 K), and resistance, respectively.

Then, we adjusted the B value of thermistor 2 to make its measured temperature as close as possible to the one monitored by thermistor 1. Table S1 shows the correlation between R_0, B, and a

temperature difference of thermistors 1 and 2. Even when the B value was adjusted, thermistor 2 showed a temperature difference of 0.02 K relative to thermistor 1.

Table S1. Correlation between R_0, B, and a temperature difference between the thermistors 1 and 2.

Thermistor	Resistance of the circuit R_0 (Ω)	Adjusted B value	Temperature difference relative to thermistor 1 (K)
1	10,000	3994	0
2	10,000	3992	0.015

S2 Measurement of the temperature at the rear side of each electrode

We utilized thermistors 1 and 2, which were calibrated according to Supplemental text S1, to measure the temperature at the rear side of each electrode. Since thermistors have always some inaccuracies and fluctuations in temperature measurements, we measured the temperatures at the rear side of each electrode at least four times to minimize the inherent measurement errors and fluctuations. Detailed procedure is as follows. First, we connected thermistor 1 to the rear side of the MA or CB electrode and thermistor 2 to the control electrode, and then measured the difference of their temperature twice. After that, we switched the two thermistors and again measured the temperature difference twice. Finally, we averaged these four data.

We adopted this measurement procedure to measure the temperature at the rear side of each electrode using thermistors 1 and 2. Based on this method, we believe that the order of the temperature difference between the MA or CB electrodes and the control electrode accurately reflects the temperature order of the MA and CB electrodes.

Table S2. Measured film thickness of the CB layer of 10 μm, 30 μm, 60 μm and 100 μm. n indicates the measurement number of the sample.

Sample name	Estimated film thickness of the CB layer (μm)	Measured film thickness of the CB layer (μm)
10 μm CB	10	9.40 ± 0.4 (n = 8)
40 μm CB	40	40.8 ± 0.6 (n = 9)
60 μm CB	60	63.9 ± 0.8 (n = 9)
100 μm CB	100	103 ± 1.0 (n = 7)

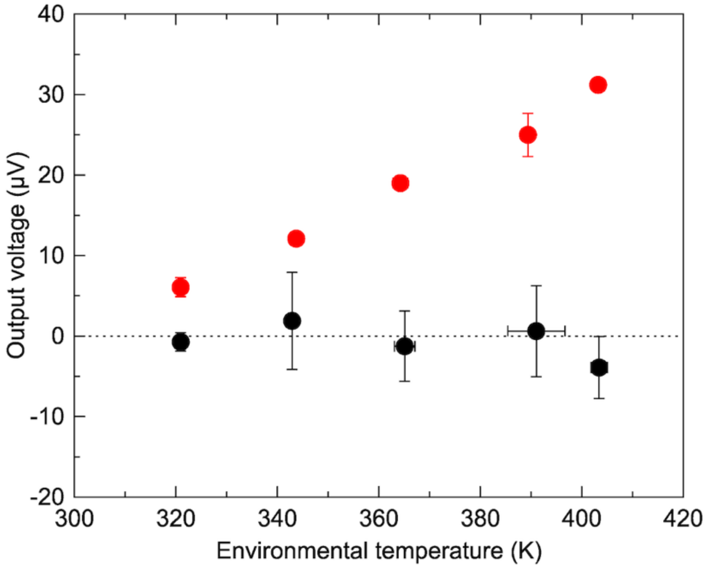

Fig. S1 Dependence of the output voltages generated on the MA device (red) and a control device (black) on the measured environment temperatures.

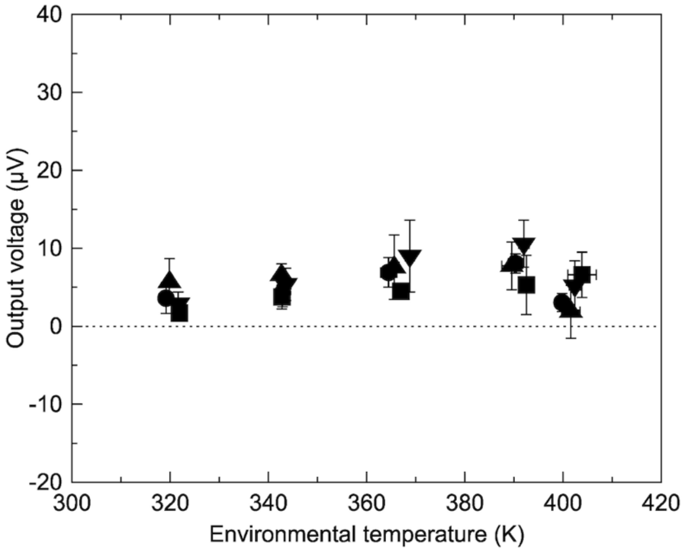

Fig. S2 Dependence of the output voltages generated on the CB device with various thickness of 10 (■), 40 (●), 60 (▲), and 100 (▼) μm on the measured environment temperatures. The error bars in the y-axis indicate the standard deviation of the measured output voltages.

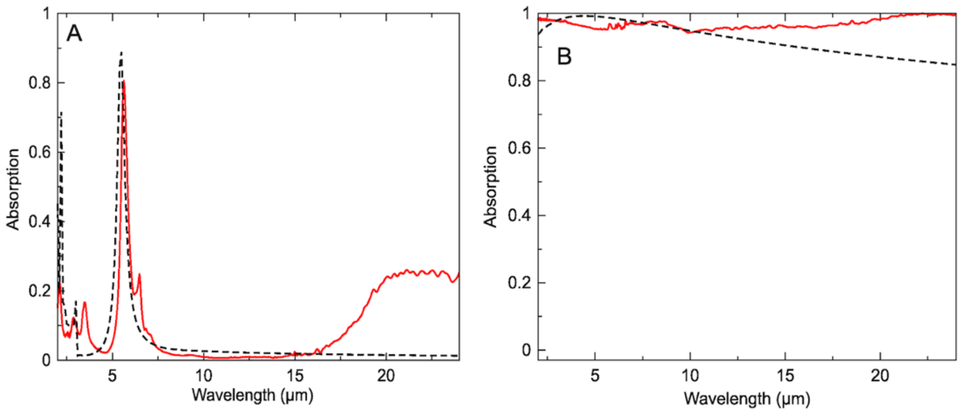

Fig. S3 Measured (red lines) and calculated (dashed lines) absorption spectrum of (A) MA (310 nm) and (B) CB (100 μm) electrodes. The reflection spectra of the samples were measured by microscopic Fourier transform infrared spectrometer (FT/IR-6300, VIRT-3000, JASCO Corporation). The reference spectrum was measured on a bare copper plate. The absorption spectra were calculated subtracting measured reflectance from 1.0.

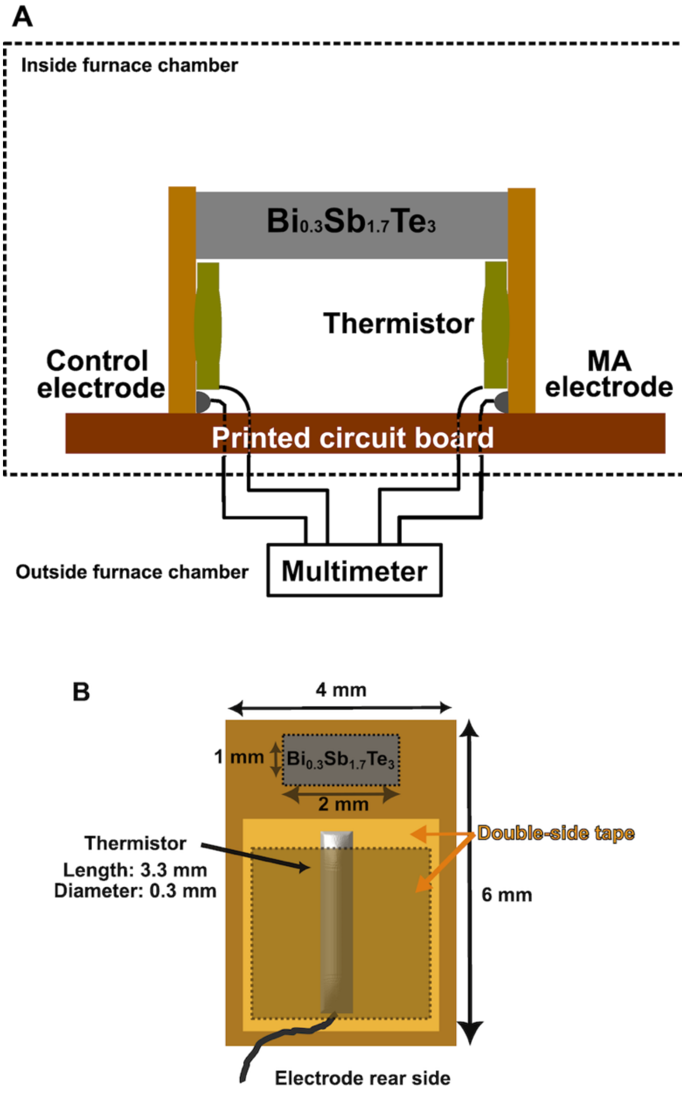

Fig. S4 Experimental setup for measuring the temperatures of the electrode rear side. The MA electrode was exchanged by the CB electrode. Schematics of (A) experimental setup in the X-Z view and (B) arrangements of the $Bi_{0.3}Sb_{1.7}Te_3$ thermoelectric element (1×2 mm^2) and a thermistor (3.3 mm $\times$ 0.3 mm) on the rear side of the Cu electrode. The thermistor was sandwiched between a Kapton double-side tape to be attached on the rear side of the Cu electrode. In the experiment, the thermistor was fully covered by the top Kapton tape.
